# Incidence of and risk factors for new-onset deep venous thrombosis after intertrochanteric fracture surgery

**DOI:** 10.1038/s41598-021-96937-w

**Published:** 2021-08-27

**Authors:** Kuo Zhao, Junzhe Zhang, Junyong Li, Hongyu Meng, Zhiyong Hou, Yingze Zhang

**Affiliations:** 1 Department of Orthopaedic Surgery, The Third Hospital of Hebei Medical University, No. 139 Ziqiang Road, Shijiazhuang, 050051 Hebei People’s Republic of China; 2 Key Laboratory of Biomechanics of Hebei Province, Shijiazhuang, 050051 Hebei People’s Republic of China; 3 Orthopaedic Research Institution of Hebei Province, Shijiazhuang, 050051 Hebei People’s Republic of China; 4 NHC Key Laboratory of Intelligent Orthopaedic Equipment (The Third Hospital of Hebei Medical University), Shijiazhuang, People’s Republic of China; 5 Chinese Academy of Engineering, Beijing, 10088 People’s Republic of China

**Keywords:** Biological techniques, Genetics

## Abstract

This study aimed to investigate the incidence of and risk factors for postoperative new-onset deep venous thrombosis (PNO-DVT) following intertrochanteric fracture surgery. Information on 1672 patients who underwent intertrochanteric fracture surgery at our hospital between January 2016 and December 2019 was extracted from a prospective hip fracture database. Demographic information, surgical data, and preoperative laboratory indices were analysed. Receiver operating characteristic curve analysis, univariate analyses and binary logistic regression analyses were performed. The incidences of postoperative deep venous thrombosis (DVT) and PNO-DVT in inpatients after intertrochanteric fracture surgery were 11.5% (202 of 1751 patients) and 7.4% (123 of 1672 patients), respectively. PNO-DVT accounted for 60.9% of postoperative DVT. Additionally, there were 20 cases of central thrombosis (16.3%), 82 cases of peripheral thrombosis (66.7%), and 21 cases of mixed thrombosis (17.1%). In addition, 82.1% of PNO-DVTs were diagnosed within 8 days after surgery. The multivariate analysis revealed that age > 70 years, duration of surgery (> 197 min), type of anaesthesia (general), and comorbidities (≥ 3) were independent risk factors for the development of PNO-DVT after intertrochanteric fracture surgery. This study demonstrated a high incidence of PNO-DVT in inpatients after intertrochanteric fracture surgery. Therefore, postoperative examination for DVT should be routinely conducted for patients.

## Introduction

Venous thromboembolism (VTE) has become the third leading vascular disease following acute myocardial infarction and stroke, and its incidence increases with age^1^. VTE is not only associated with increasing morbidity and mortality but also correlated with a greater economic burden^2,3^. Deep venous thrombosis (DVT), one main manifestation of venous thromboembolism (VTE), is a serious complication that is closely associated with increased morbidity and mortality after intertrochanteric fractures^4^. Previous studies have reported that the incidence of perioperative DVT due to hip fractures, including femoral neck fractures and intertrochanteric fractures, ranges from 11.1 to 34.98%^1,5–7^. Some studies have also reported that the prevalence of DVT is as high as 62% in patients with hip fractures when the time to surgery is delayed by more than 2 days^8^. Most of these studies were conducted to detect the incidence of and risk factors for preoperative DVT in patients with hip fractures and combined the rates of DVT for femoral neck fractures and intertrochanteric fractures. Park et al.^9^ found that the prevalence of preoperative DVT following hip fractures was 18.4% (56 of 305 patients). A 16.3% incidence of preoperative DVT following hip fractures has been reported by Luksameearunothai et al.^10^.

It has been reported that postoperative DVT can increase patient mortality^11^. However, limited studies have been conducted to detect the incidence of postoperative DVT, especially postoperative new-onset DVT (PNO-DVT), after hip fractures. Song et al.^6^ reported that the prevalence of postoperative DVT following hip fractures was 32.8% (39 in 119 patients). Zhang et al. investigated the prevalence of preoperative and postoperative DVT in patients with hip fractures and found that the incidence of postoperative DVT was 57.23%^6^. These studies did not explore the incidence of or risk factors for PNO-DVT, which is important for the perioperative management of intertrochanteric fractures. Furthermore, no study has investigated the prevalence of or risk factors for PNO-DVT in inpatients with intertrochanteric fractures alone. Previous studies have reported several risk factors associated with the rate of DVT after hip fractures, including age, female sex, cardiovascular disease, pulmonary disease, cancer, previous hospitalization for VTE, and type of anaesthesia^1,12,13^. Recent studies have found that the time from injury to surgery, anaemia, fibrinogen, D-dimer, and blood loss are associated with the incidence of DVT after hip fractures^6,9,14^. Park et al. found that high-energy injuries were associated with the development of DVT after hip fractures^15^.

Few studies have investigated the risk factors for PNO-DVT in intertrochanteric fracture patients. The present study aimed to detect the incidence of and risk factors for PNO-DVT in inpatients with intertrochanteric fractures.

## Patients and methods

### Patients

**Figure 1:**
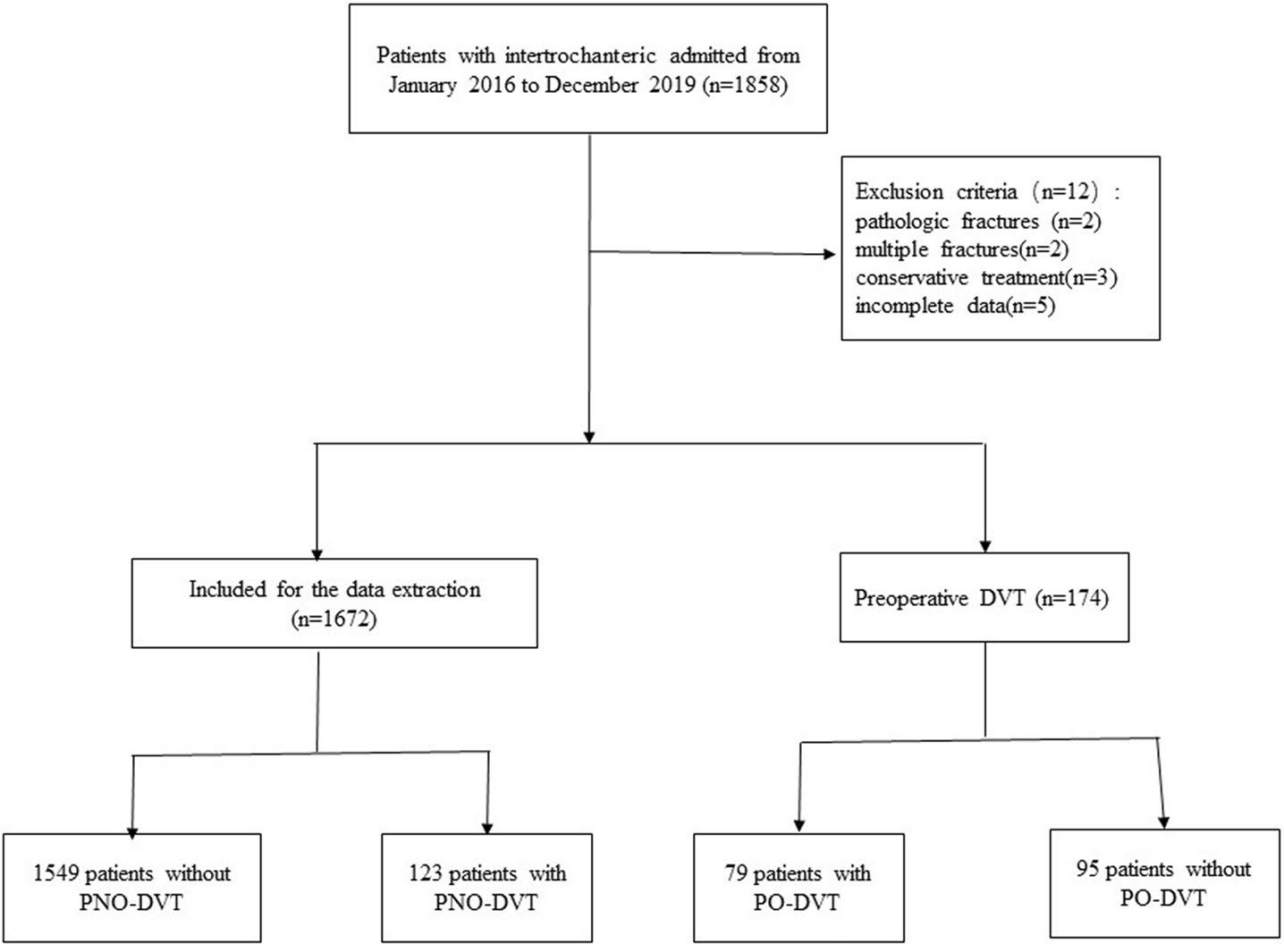
The flow chart for the selection of study participants.

This study was a prospective study and was performed in a level I trauma centre of a tertiary university hospital. Data from a total of 1672 patients who underwent intertrochanteric fracture surgery in our hospital from January 2016 to December 2019 were extracted from a prospective intertrochanteric fracture database and analysed according to the exclusion criteria (Fig. 1). The exclusion criteria of this study were patients with (1) conservative treatment; (2) preoperative DVT; (3) pathologic fracture; (4) multiple fractures; and (5) incomplete data. The Institutional Review Board of Third Hospital of Hebei Medical University approved our study. This study followed the principles outlined in the Helsinki Declaration, and informed consent was obtained from all patients.

### Methods

Patients with intertrochanteric fractures were conventionally injected with subcutaneous low-molecular-weight heparin sodium (LMWHS, 4250 IU, once daily) during their hospital stay, and LMWHS was stopped at least 12 h prior to surgery and restarted at least 12 h after surgery^16,17^. Mechanical prophylaxis was performed by an intermittent foot pump during hospitalization. Colour Doppler ultrasound was used to detect the presence of DVT in both lower limbs at hospitalization, after the operation (1–2 days after surgery) and before discharge. If the surgery was delayed more than 72 h, ultrasound examination was performed again before the operation. Examination was required every 3 days postoperatively until discharge from the hospital. The diagnosis of DVT was made by sonographers, and the diagnostic criteria were based on the Robinov group’s criteria^18^. PNO-DVT in this study was defined as the in-hospital postoperative new-onset DVT which was occurring during hospitalization after operation. We recorded the types of DVT, which were divided into central-type thrombosis, peripheral-type thrombosis, and mixed-type thrombosis. The central type included thrombosis in the iliac, superficial femoral, femoral and popliteal veins, occurring proximal to the knee. The peripheral type referred to thrombosis in the posterior tibial and peroneal veins. The mixed type was defined as thrombosis involving both types. The time of thrombus formation was also reviewed.

We selected approximately 80 variables that might be potential risk factors for the development of DVT after intertrochanteric fractures, including demographic and fracture characteristics, laboratory indices, and surgical data. The demographic characteristics included age, sex, body mass index (BMI), residential location (rural or urban), medical complications, smoking history, and disease (e.g., cancer). The medical comorbidities were hypertension, cardiovascular disease (coronary heart disease and arrhythmia), cerebrovascular disease (haemorrhagic and ischaemic encephalopathy), chronic respiratory disease, (diabetes mellitus, chronic bronchitis, chronic obstructive pulmonary disease, and bronchiectasis), liver disease (viral hepatitis and liver cirrhosis), renal disease (glomerulonephritis and chronic renal failure), and rheumatologic disease. The number of medical comorbidities was recorded as 0, 1–2, or ≥ 3. The American Society of Anesthesiologists score (ASA score) of each patient was also obtained, and the cases were divided into scores of 1–2 and 3–4. The fracture characteristics and surgical variables included the injury mechanism, duration of surgery, anaesthesia method, and implant type (intramedullary or extramedullary device). The laboratory indices were preoperative laboratory indices that were measured at the time of admission.

### Statistical analysis

All patients were divided into two groups: the DVT group and the without-DVT group. Receiver operating characteristic (ROC) analysis was conducted to identify the optimum cut-off value for continuous variables, including age, time to surgery, duration of surgery, and variables (e.g., BMI). Continuous variable data are presented as the mean ± standard deviation and were analysed by either Student’s *t* test or the Mann–Whitney *U* test as appropriate. Nonnormally distributed variables are reported as median values with quartiles. Categorical variables are presented as the frequency and percentage and were tested by the Chi-square or Fisher’s exact test. Binary logistic regression modelling was conducted to distinguish the independent predictors of DVT according to the results of the univariate analysis. SPSS v23.0 software (IBM, Armonk, NY, USA) was used for all statistical analyses. Statistical significance was defined as p < 0.05.

## Results

### Clinical parameters

**Figure 2:**
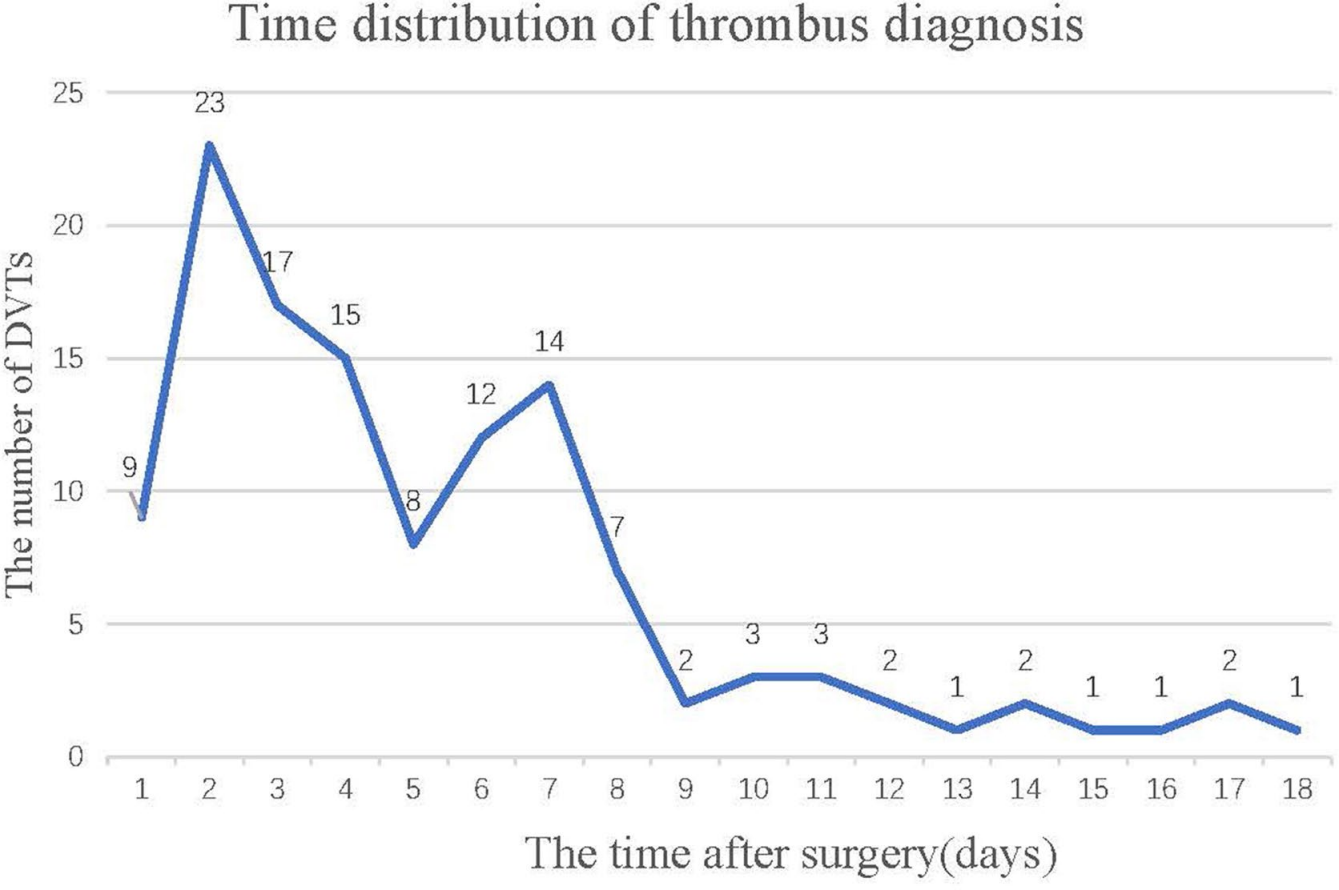
The diagnosis time of PNO-DVT in patients.

A total of 1672 patients with intertrochanteric fractures were analysed in our study. The incidences of postoperative DVT and PNO-DVT in inpatients after intertrochanteric fracture surgery were 11.5% (202 of 1751 patients) and 7.4% (123 of 1672 patients), respectively. PNO-DVT accounted for 60.9% of postoperative DVT. Additionally, there were 20 cases of central thrombosis (16.3%), 82 cases of peripheral thrombosis (66.7%), and 21 cases of mixed thrombosis (17.1%). The detection time of DVT was shown in Fig. 2, and 82.1% of DVTs were diagnosed within 8 days after surgery. Moreover, the results showed that 7 proximal PNO-DVT patients were treated with inferior vena cava filter placement, and the others, including proximal PNO-DVT, distal PNO-DVT and mixed PNO-DVT patients, received anticoagulation therapy (LMWH sodium, 4250 IU every 12 h). Ultimately, complete recanalization or partial recanalization was observed in all of the patients.

The results of the ROC curve analysis are shown in Table 1. The comparison of demographics and fracture characteristics between the two groups is shown in Table 2. There were 965 females and 707 males. The average age of all patients was 73.2 ± 14.4 years. Significant differences were found between the two groups in patients aged > 70 years (68.3% in the without-DVT group vs 78.9% in the DVT group, p = 0.015). There were more patients with high-energy injuries in the DVT group than in the without-DVT group, but no significant difference was found between the groups in terms of the injury mechanism (p = 0.346). Significant differences in terms of traumatic brain injury and comorbidities (no.) were found between the two groups. The mean intraoperative blood loss in the DVT group was 323.6 ± 466.52 ml, which was more than that in the without-DVT group. A comparison of preoperative laboratory indicators between the two groups is shown in Table 3, and no significant difference was found between the two groups. A comparison of surgical data between the two groups is shown in Table 4. Significant differences were found between the two groups in terms of spinal anaesthesia, implant and duration of surgery. In addition, the other data were comparable between them.

**Table 1:** Optimum cut-off value of continuous variables detected by the ROC analysis.

Variables	Cut-off value	Area under the ROC curve (AUC)	P value	95% CI
Age (years)	70	0.560	0.025	0.510–0.609
Time to surgery (days)	2	0.514	0.592	0.462–0.566
Duration of surgery (min)	197	0.505	0.853	0.450–0.560

*ROC* receiver-operating characteristic, *CI* confidence interval.

**Table 2:** Comparison of demographics and fracture characteristics between the two groups.

Variables	Overall (N = 1672)	Without DVT (N = 1549)	With DVT (N = 123)	P value
Age (> 70, years), n (%)	1155 (69.1)	1058 (68.3)	97 (78.9)	0.015
Gender (male), n (%)	707 (42.3)	656 (42.3)	51 (41.5)	0.848
Residential location (urban), n (%)	749 (44.8)	692 (44.7)	57 (46.3)	0.720
Hypertension, n (%)	746 (44.6)	694 (44.8)	52 (42.3)	0.587
Diabetes, n (%)	342 (20.5)	313 (20.2)	29 (23.6)	0.372
Cerebrovascular disease, n (%)	515 (30.8)	471 (30.4)	44 (35.8)	0.215
Cardiovascular disease, n (%)	551 (33.0)	502 (32.4)	49 (39.8)	0.009
Chronic respiratory disease, n (%)	83 (5.0)	79 (5.1)	4 (3.3)	0.364
Smoking history, n (%)	113 (6.8)	107 (6.9)	6 (4.9)	0.388
Preoperative systemic infection, n (%)	41 (2.5)	41 (2.6)	0 (0.0)	0.068
Tumors, n (%)	36 (2.2)	33 (2.1)	3 (2.4)	0.820
Traumatic brain injury, n (%)	20 (1.2)	16 (1.1)	4 (3.3)	0.029
Liver disease, n (%)	36 (2.2)	33 (2.1)	3 (2.4)	0.820
Renal disease, n (%)	48 (2.9)	47 (3.0)	1 (0.8)	0.156
Rheumatoid diseases, n (%)	21 (1.3)	20 (1.3)	1 (0.8)	0.647
Previous surgical history	258 (15.4)	234 (15.1)	24 (19.5)	0.193
**Comorbidities (no.), n (%)**				0.004
0	323 (19.3)	309 (19.9)	14 (11.4)	
1–2	758 (45.3)	708 (45.7)	50 (40.7)	
≥ 3	591 (35.3)	532 (34.3)	59 (48.0)	
ASA3–4, n (%)	767 (45.9)	707 (45.9)	60 (48.8)	0.891
**BMI, n (%)**				0.664
< 18.5	93 (5.6)	88 (5.7)	5 (4.1)	
18.5–23.9	1009 (60.3)	939 (60.6)	70 (56.9)	
24–27.9	428 (25.6)	394 (25.4)	34 (27.6)	
28–31.9	115 (6.9)	104 (6.7)	11 (8.9)	
≥ 32	27 (1.6)	24 (1.5)	3 (2.4)	
Injury mechanism (high energy), n (%)	139 (8.3)	126 (8.1)	13 (10.6)	0.346
Side (left), n (%)	809 (48.4)	707 (51.5)	66 (53.7)	0.637

*ASA* American Society of Anesthesiologists, *BMI* body mass index.

**Table 3:** Comparison of preoperative laboratory indicators between the two groups.

Variables	Without POP (N = 1945)	With POP (N = 53)	P value
TP < 65 g/L, n (%)	1185 (82.2)	47 (88.7)	0.222
ALB < 35 g/L, n (%)	975 (67.6)	37 (69.8)	0.737
**GLOB (references 20–40 g/L), n (%)**			0.693
< 20	228 (15.8)	7 (13.2)	
> 40	12 (0.8)	0 (0.0)	
**A/G (references 1.2–2.4), n (%)**			0.676
< 1.2	314 (21.8)	13 (24.5)	
> 2.4	16 (1.1)	0 (0.0)	
**ALT (references 9–50 U/L), n (%)**			0.431
< 9	162 (11.2)	8 (15.1)	
> 50	72 (5.0)	1 (1.9)	
**AST (references 15–40 U/L), n (%)**			0.807
< 15	269 (18.7)	9 (17.0)	
> 40	140 (9.7)	4 (7.5)	
TBIL (> 26), n (%)	192 (13.3)	9 (17.0)	0.442
DBIL (> 6), n (%)	648 (44.9)	30 (56.6)	0.145
IBIL (> 14), n (%)	242 (16.8)	10 (18.9)	0.061
**ALP (references 45–125 U/L), n (%)**			0.720
< 45	169 (11.7)	7 (13.2)	
> 125	92 (6.4)	2 (3.8)	
**GGT (references 10–60 U/L), n (%)**			0.936
< 10	83 (5.8)	3 (5.7)	
> 60	116 (8.0)	5 (9.4)	
**CHE (references 5–12 U/L), n (%)**			0.254
< 2	627 (43.5)	29 (54.7)	
> 12	5 (0.3)	0 (0.0)	
**TBA (references 1–10 μmol/L), n (%)**			0.411
< 1	91 (6.3)	3 (5.7)	
> 10	164 (11.4)	3 (5.7)	
HCRP (> 8 mg/L), n (%)	1210 (83.9)	45 (84.9)	0.846
CK (> 310 U/L), n (%)	297 (20.6)	11 (20.8)	0.978
CKMB (> 20 U/L), n (%)	219 (15.2)	14 (26.4)	0.027
LDH (> 250 U/L), n (%)	461 (32.0)	16 (30.2)	0.785
TC (> 5.2 μmol/L), n (%)	95 (6.6)	2 (3.8)	0.414
TG (> 1.7 μmol/L), n (%)	99 (6.9)	4 (7.5)	0.847
**Na (references 137–147 mmol/L), n (%)**			0.471
< 137	663 (46.0)	26 (49.1)	
> 147	9 (0.6)	1 (1.9)	
**K (references 3.5–5.3 mmol/L), n (%)**			0.610
< 3.5	205 (14.2)	6 (11.3)	
> 5.3	16 (1.1)	0 (0.0)	
**CL (references 99–110 mmol/L), n (%)**			0.050
< 99	225 (15.6)	65 (4.5)	
> 110	65 (4.5)	6 (11.3)	
**TCO2 (references 20–30 mmol/L), n (%)**			0.889
< 20	69 (4.8)	3 (5.7)	
> 30	721 (5.0)	2 (3.8)	
GLU (> 6.1), n (%)	868 (60.2)	34 (64.2)	0.761
UREA (> 8), n (%)	327 (22.8)	15 (28.3)	0.320
**CREA (references 57–97 mmol/L), n (%)**			0.563
< 57	623 (43.2)	20 (37.7)	
> 97	92 (6.4)	5 (9.4)	
**UA (references 208–428 mmol/L), n (%)**			0.302
< 208	729 (40.6)	22 (41.5)	
> 428	44 (3.1)	3 (5.7)	
**CA (references 2.11–2.52 mmol/L), n (%)**			0.600
< 2.11	761 (52.8)	31 (58.5)	
> 2.52	12 (0.8)	0 (0.0)	
**P (references 0.85–1.51 mmol/L), n (%)**			0.579
< 0.85	185 (12.8)	9 (17.0)	
> 1.51	50 (3.5)	1 (1.9)	
**Mg (references 0.75–1.02 mmol/L), n (%)**			0.671
< 0.75	167 (11.6)	44 (3.1)	
> 1.02	8 (15.1)	1 (1.9)	
**BNP (ng/L), n (%)**			< 0.001
< 75	558 (38.7)	9 (17.0)	
> 75	416 (28.8)	30 (56.6)	
Unknown	468 (32.5)	14 (26.4)	
**WBC (references 3.5–9.5 × 10**^**9**^**/L), n (%)**			0.307
< 3.5	10 (0.7)	0 (0.0)	
> 9.5	513 (35.6)	24 (45.3)	
**NEU (references 2.8–6.3 × 10**^**9**^**/L), n (%)**			0.071
< 1.8	3 (0.3)	0 (0.0)	
> 6.3	749 (51.9)	36 (67.9)	
**LYM (references 1.1–3.2 × 10**^**9**^**/L), n (%)**			0.818
< 1.1	752 (52.1)	29 (54.7)	
> 3.2	8 (0.6)	0 (0)	
**MON (references 0.1–0.6 × 10**^**9**^**/L), n (%)**			0.520
< 0. 1	3 (0.2)	0 (0)	
> 0.6	870 (60.3)	36 (67.9)	
**EOS (references 0.02–0.05 × 10**^**9**^**/L), n (%)**			0.052
< 0.02	373 (25.9)	9 (17.0)	
> 0.52	4 (0.3)	1 (1.9)	
BAS (> 0.06), n (%)	83 (5.8)	3 (5.7)	0.977
RBC (> 5.8), n (%)	94 (6.5)	4 (7.5)	0.766
**NEU% (references 45–75%), n (%)**			0.650
< 45	3 (0.2)	0 (0.0)	
> 75	865 (60.0)	35 (66.0)	
LYM% (> 5.2), n (%)	1378 (95.6)	50 (94.3)	0.673
**MON% (references 3–10%), n (%)**			0.638
< 3	24 (1.7)	0 (0.0)	
> 10	343 (23.8)	13 (24.5)	
BAS% (> 1%), n (%)	10 (0.7)	11 (0.7)	0.318
HGB (< 110/120 g/L), n (%)	1334 (92.5)	50 (94.3)	0.618
**MCV (references 82–100 fL), n (%)**			0.086
< 82	31 (2.1)	0 (0.0)	
> 100	110 (7.6)	8 (15.1)	
**MCH (references 27–34 pg), n (%)**			0.189
< 27	38 (2.6)	0 (0.0)	
> 34	92 (6.4)	6 (11.3)	
**MCHC (references 316–354 g/L), n (%)**			0.074
< 316	38 (2.6)	3 (5.7)	
> 354	61 (4.2)	5 (9.4)	
**RDW (references 11.6–16.5%), n (%)**			0.912
< 11.6	5 (0.3)	0 (0.0)	
> 16.5	136 (9.4)	5 (9.4)	
**PLT (references 125–350 × 10**^**9**^**/L), n (%)**			0.996
< 125	135 (9.4)	5 (9.4)	
> 250	86 (6.0)	3 (5.7)	
**MPV (references 7.4–11.0 fL), n (%)**			0.246
< 7.4	227 (15.7)	12 (22.6)	
> 11.0	49 (3.4)	3 (5.7)	
**PCT (references 0.16–0.43%), n (%)**			0.010
< 0.16	937 (65.0)	34 (64.2)	
> 0.43	7 (0.5)	2 (3.8)	
**PDW (references 12.0–18.1%), n (%)**			0.934
< 7.4	130 (9.0)	4 (7.5)	
> 11.0	26 (1.8)	1 (1.9)	
PT (> 12.5 S), n (%)	333 (23.1)	10 (18.9)	0.246
PTA (< 80%), n (%)	175 (12.1)	7 (13.2)	0.873
INR (> 1.4%), n (%)	11 (0.8)	1 (1.9)	0.368
**APTT (references 28–42 S), n (%)**			0.934
< 28	500 (34.7)	24 (45.3)	
> 42	20 (1.4)	0 (0.0)	
**APTT-R (references 0.7–1.3), n (%)**			0.528
< 0.7	18 (1.2)	0 (0.0)	
> 1.3	16 (1.1)	0 (0.0)	
**TT (references 14–21 S), n (%)**			0.696
< 14	1026 (71.2)	35 (66.0)	
> 21	151 (10.5)	6 (11.3)	
**FIB (reference 2.0–4.4 mg/L), n (%)**			0.708
< 2.0	16 (1.1)	0 (0.0)	
> 4.4	350 (24.3)	14 (26.4)	
**ATIII (reference 80–120%), n (%)**			0.916
< 80	295 (20.5)	12 (22.6)	
> 120	33 (2.3)	1 (1.9)	
D-Dimer (> 2.26 mg/L), n (%)	571 (39.6)	34 (64.2)	< 0.001

*ASA* American Society of Anesthesiologists, *BMI* body mass index, *TP* total protein, *ALB* albumin, *GLOB* globulin, *A/G values* albumin/globulin, *ALT* alanine transaminase, *AST* aspartate aminotransferase, *TBIL* total bilirubin, *DBIL* direct bilirubin, *IBIL* indirect bilirubin, *ALP* alkaline phosphatase, *GGT* γ-glutamyl transpeptidase, *CHE* cholinesterase, *TBA* total bile acid, *HCRP* hypersensitive c-reactive protein, *LDH* lactate dehydrogenase, *CREA* creatinine, *UA* uric acid, *CA* calcium, *P* phosphorus, *Mg* magnesium, *BNP* brain natriuretic peptide, *WBC* white blood cell, *NEUT* neutrophile, *LYM* lymphocyte, *MON* mononuclear cell, *EOS* eosinophilic granulocyte, *BAS* basophilic granulocyte, *RBC* red blood cell, reference range: female, 3.5–5.0 × 10^12^/L; males, 4.0–5.5 × 10^12^/L. *HGB* hemoglobin, reference range: females, 110–150 g/L; males, 120–160 g/L, *HCT* haematocrit, 40–50%, *MCV* mean corpuscular volume, *MCH* mean corpuscular hemoglobin, *MCHC* mean corpuscular hemoglobin concentration, *RDW* red blood cell distribution width, *PLT* platelet, 100–300 × 10^9^/L, *MPV* mean platelet volume, *PCT* procalcitonin, *pdw* platelet distribution width, *PT* prothrombin time, *PTA* prothrombin activity, *INR* international normalized ratio, *APTT* activated partial thromboplastin time, *APTT-R* activated partial thromboplastin time ratio, *TT* thrombin time, *TT-R* thrombin ratio, *FIB* fibrinogen, *ATIII* antithrombin III.

**Table 4:** Comparison of surgical data between the two groups.

Variables	Without DVT (N = 1549)	With DVT (N = 123)	P value
Intraoperative blood loss (ml), mean (SD)	277.5 (259.87)	323.6 (466.52)	0.079
Intraoperative blood transfusion (ml), mean (SD)	143.8 (349.53)	102.85 (223.82)	0.201
Time to surgery (> 2 day), n (%)	1261 (81.9)	102 (82.9)	0.772
Type of anesthesia (general), n (%)	648 (41.8)	66 (53.7)	0.011
Implant, n (%)			0.025
Intramedullary devices	1438 (92.8)	122 (99.2)	
Extramedullary devices	111 (7.2)	1 (0.8)	
Duration of surgery (> 197 min), n (%)	90 (5.8)	15 (12.2)	0.005

### Risk factors for PNO-DVT

In the univariate analysis, age > 70 years, traumatic brain injury, comorbidities (no.), P, type of anaesthesia, implant and duration of surgery exhibited significant differences between the two groups. All the above factors were analysed in the multivariate analysis, and the results are shown in Table 5. Age > 70 years (OR = 1.832, p = 0.016), duration of surgery > 197 min (OR = 3.733, p = 0.000), general anaesthesia (OR = 1.558, p = 0.023) and number of comorbidities ≥ 3 (OR = 2.196, p = 0.015) were independent factors for increasing the risk of PNO-DVT.

**Table 5:** OR, 95% CI, and P value for independent risk factors in the multivariable logistic regression analysis of PNO-DVT.

Variable	OR	95%CI	P value
Age (> 70 years)	1.832	1.117–3.002	0.016
Duration of surgery (> 197 min)	3.733	1.955–7.219	0.000
Type of anesthesia (general)	1.558	1.064–2.281	0.023
**Comorbidities (no.)**			
0			0.026
1–2	1.479	0.787–2.781	0.224
≥ 3	2.196	1.163–4.416	0.015

*OR* odds radio, *CI* confidence interval, *CKMB* creatine phosphokinase isoenzyme, *BNP* brain natriuretic peptide.

## Discussion

Although pharmacological prophylaxis is recommended in hip fracture patients, the incidence of DVT remains high. The incidences of postoperative DVT and PNO-DVT in inpatients after intertrochanteric fracture surgery were 11.5% (202 of 1751 patients) and 7.4% (123 of 1672 patients), respectively. Patients with PNO-DVT accounted for 60.9% of those with postoperative DVT. A total of 82.1% of DVTs were diagnosed within 8 days after surgery. However, the diagnosis of DVT might be delayed because the examinations were performed every 3–5 days after surgery. In addition, the results of multivariate logistic regression analysis demonstrated that age > 70 years, duration of surgery > 197 min, general anaesthesia and number of comorbidities ≥ 3 were independent risk factors for the development of PNO-DVT.

The 11.5% incidence of postoperative DVT was similar to that of DVT in previous studies. In their study of DVT in hip fractures, Eriksson et al.^19^ found that the prevalence of DVT after hip fractures was 14% within postoperative day 11. Wang et al.^20^ detected the incidence of postoperative DVT in patients with intertrochanteric fractures. They found that the prevalence of DVT after intertrochanteric fracture surgery was 9.94% in 311 patients.

Patients with postoperative DVT could be divided into those with preoperative DVT and those without preoperative DVT, and those without preoperative DVT were defined as PNO-DVT in this study. In the present study, the incidence of PNO-DVT was 7.4% (123 of 1672 patients), which accounted for 60.9% of postoperative DVT. In addition, 39.1% of those patients had preoperative DVT, which was excluded from the statistical analysis. For patients with preoperative DVT, the dosage of LMWH and physical prophylaxis might be different from those of patients without preoperative DVT. In addition, their coagulation function might vary. All of the above might result in possible differences in risk factors for postoperative DVT between patients with PNO-DVT and patients with preoperative DVT. Moreover, a better understanding of the risk factors for PNO-DVT is conducive to taking more measures to prevent the development of PNO-DVT.

Four independent predictive factors for PNO-DVT in inpatients after intertrochanteric fracture surgery were identified in this study. As an independent risk factor for DVT, advanced age has been reported in previous studies. In this study, age > 70 years was a cut-off value for the development of PNO-DVT detected by ROC curve analysis. Shahi et al. reported a risk factor for the development of in-hospital VTE after hip surgery^21^. These researchers demonstrated that age > 70 years (OR: 1.3, 95%, CI: 1.1–1.4) was an independent factor for increasing the risk of developing in-hospital VTE, which was consistent with our study. Park et al. also reported that age > 60 was an independent risk factor for the development of DVT^12^. Advanced age has always been associated with frailty and additional comorbidities. Frailty is a common status in patients with intertrochanteric fractures, especially in patients with advanced age, and can seriously affect their quality of life^22^. Anaemia is a common condition in patients with advanced age that has been demonstrated to increase the risk of DVT^23^. In addition, immobilization tends to be longer in patients with advanced age, which is one of the primary reasons for the development of DVT^24^.

The hypercoagulation state is well known as a main factor promoting thrombosis^2^. Surgery is a significant factor for the formation of DVT after acute trauma in terms of the introduction of the hypercoagulability state^5,25^. It has been reported that approximately 15% of all VTEs are surgery-related^26^. The surgery duration was 197 min according to the results of the ROC curve analysis. Multivariate logistic regression analyses revealed that a duration of surgery > 197 min was an independent risk factor for PNO-DVT. Blood loss increased with a prolonged duration of surgery. Riha et al. found that blood loss was an important factor in promoting the hypercoagulability state in their study^27^. Their study showed that blood loss was associated with an increase in the risk of DVT. Zhang et al. studied the incidences of DVT before and after surgery in in-hospital patients with hip fractures and found that blood loss was correlated with the formation of postoperative DVT^6^. Therefore, a longer duration of surgery might be associated with the development of DVT, leading to an increased level of coagulation.

The optimal anaesthetic modality in hip fracture surgery remains controversial. The choice of anaesthesia modality in hip fracture surgery often depends on the preference of the surgeon or the anaesthesiologist^28^. Previous studies have reported that the anaesthesia modality in hip fracture surgery plays an important role in the occurrence of postoperative complications^29,30^. Some studies have demonstrated that spinal anaesthesia is superior to general anaesthesia in preventing DVT, urinary tract infection, blood loss, superficial wound infection and overall complications^13,31^. The results of this study were the same as those of a previous study on the association between anaesthesia and DVT. The percentage of patients with general anaesthesia was 53.7% (66 of 123 patients) in the DVT group, whereas the percentage of patients in the without-DVT group was 41.8%. Significant differences in anaesthesia modality were found between the two groups (p = 0.011). In addition, the present study demonstrated that the risk for developing DVT in patients with general anaesthesia after intertrochanter fracture surgery was increased 1.558-fold compared with that in patients with spinal anaesthesia. The explanation for this phenomenon might be that general anaesthesia increases the length of hospital stay^32^. Further studies should be conducted to explore the specific mechanism of anaesthesia and DVT.

There were some strengths in our study. Few studies have investigated the risk factors for PNO-DVT, and this study excluded patients with preoperative DVT. Moreover, the data of this study were based on a prospective database, and approximately 80 factors were analysed in this study. All of the above would help increase the reliability and accuracy of the results in the present study. However, our study did have some limitations. First, all the data were extracted from one hospital. Additionally, this study was a single-centre study, which was limited by its inherent defects. Further multicentre randomized controlled trials are warranted. Second, some comorbidities, such as varicose veins and defects of the coagulation system, were not discussed in our study. Third, one limitation of this study was that the recent use of antiplatelet drugs (within 1 week before injury) was not analysed. A previous study reported that the recent use of antiplatelet drugs (within 1 week before injury) could decrease the incidence of preoperative DVT in femoral neck fracture. However, the effect of the recent use of antiplatelet drugs on postoperative hip fractures was not demonstrated. The potential mechanism by which antiplatelet drugs decrease the development of preoperative DVT might be associated with blood coagulation indices. Considering the potential influence of the recent use of antiplatelet drugs on PNO-DVT, we extracted the preoperative blood coagulation indices of all patients on admission. Fourth, no follow-up on patients was conducted, and the association between PNO-DVT and adverse events after surgery in the mid- or long-term could not be demonstrated.

## Conclusion

This study demonstrated a high incidence of PNO-DVT in inpatients after intertrochanteric fracture surgery. Therefore, postoperative examination for DVT should be routinely conducted for patients.
